# Subspecialty Faculty in Obstetrics and Gynecology: Distribution, Demographics, and Implications for Training and Clinical Practice

**DOI:** 10.7759/cureus.48736

**Published:** 2023-11-13

**Authors:** Morgan R Steffen, Heng Jiang, Taoyuan Beninato, Alexandra Pool, Shilpa Tummala, Emma Poulas, Mary W Kinyoun, Tyler M Muffly

**Affiliations:** 1 Obstetrics and Gynecology, University of Nebraska Medical Center, Omaha, USA; 2 Obstetrics and Gynecology, University of Colorado Anschutz Medical Campus, Aurora, USA; 3 Obstetrics and Gynecology, Denver Health and Hospital Authority, Denver, USA

**Keywords:** reproductive endocrinology and infertility, gynecologic oncology, maternal-fetal medicine, urogynecology, obstetrics and gynecology residency, subspecialist, physician workforce, medical workforce

## Abstract

Objective: The objective of this study was to quantify the subspecialist workforce involved in the clinical education of Obstetrics and Gynecology (OBGYN) residents and to provide an overview of the subspecialist faculty workforce geographic distribution and demographics.

Methods: This cross-sectional, observational study used public data collected from July 1, 2022, through August 31, 2022. A list of Obstetrics and Gynecology residency programs, their sponsoring institutions/locations, and affiliated locations was compiled from the American Medical Association's Fellowship and Residency Electronic Interactive Database. Faculty subspecialists' names were collected by manually searching each program's website. Demographics were collected from the National Plan and Provider Enumeration System. Subspecialty faculty who had completed an Obstetrics and Gynecology residency, were fellowship trained, and/or had board certification in the subspecialty were included in the study.

Results: A total of 4,659 subspecialist faculty were identified from 278 residency programs, representing 81.5% of the total subspecialist workforce in Obstetrics and Gynecology (n=5,716). Of the subspecialists identified, 2,838 were faculty at sponsoring institutions, representing 49.7% of the entire subspecialist workforce; the remainder worked with residents at affiliate locations. Our results showed 59.9% of subspecialists were female and 40.1% were male; 97.0% were allopathic subspecialists. The largest proportion of subspecialists were in the age group of 40-49 years (36.6%). Subspecialists were present in 45 states, with the exception of Alaska, Idaho, Montana, North Dakota, South Dakota, and Wyoming.

Conclusion: Most of the Obstetrics and Gynecology subspecialty workforce is involved in the clinical education of OBGYN residents, with half of the workforce on faculty at the residency program sponsor site. The subspecialty faculty workforce is primarily female, has an allopathic degree, is mid-career, and is geographically diverse.

## Introduction

Medicine has witnessed increasing subspecialization, and Obstetrics and Gynecology (OBGYN) is no exception [1]. The Accreditation Council for Graduate Medical Education (ACGME)-approved subspecialties in OBGYN include Gynecologic Oncology (GYO), Maternal-Fetal Medicine (MFM), Reproductive Endocrinology and Infertility (REI), and Female Pelvic Medicine and Reconstructive Surgery (FPMRS), now referred to as Urogynecology and Reconstructive Pelvic Surgery (URPS) [2]. Also ACGME-accredited, Complex Family Planning (CFP) is the newest American Board of Obstetrics and Gynecology (ABOG) certification [2-3]. Additionally, Minimally Invasive Gynecologic Surgery (MIGS) and Pediatric and Adolescent Gynecology (PAG) are established certifications overseen by professional organizations [4-5]. As per the ACGME Program Requirements for Graduate Medical Education in OBGYN, programs must designate a qualified “Subspecialty Faculty Educator,” who is either currently certified in the subspecialty by ABOG or possesses qualifications acceptable to the Review Committee [6]. This individual is crucial in coordinating resident education and ensuring a valuable learning experience in each subspecialty.

Exposure to subspecialty mentorship during residency has been cited as a significant factor in residents' decision to pursue subspecialization [7]. The rate of residents pursuing fellowship is growing disproportionately to the number of generalists entering the field each year [1,8]. This trend was highlighted in the most recent American College of Obstetricians and Gynecologists (ACOG) evaluation of subspecialist distribution in 2017 by Rayburn [8]. The same study noted large geographic discrepancies in subspecialty care, hypothesizing that most subspecialists work at or near university-based training sites, which are largely located in urban areas, leaving rural areas without access to subspecialty trained practitioners.

This study aims to quantify the OBGYN subspecialty workforce practicing at ACGME-affiliated residency programs. The analysis provides an insight into the size and composition of the faculty subspecialist workforce and the distribution by factors such as gender, age, and location. The OBGYN workforce census, diversity and distribution have implications for residency training programs as they work to meet ACGME program requirements and recruit residents, and also for patient care, as the diversity and distribution of the workforce increases and changes to meet the needs of the population.

## Materials and methods

Program identification

This cross-sectional study utilized publicly available data and was deemed exempt from Institutional Review Board review. The American Medical Association (AMA) Fellowship and Residency Electronic Interactive Database Access (FREIDA) was used to compile a comprehensive list of OBGYN residencies accredited by the ACGME [9]. Information on program locations (city and state) and practice settings (university-based, university-based community-affiliated, community-based) were recorded [9]. Sponsoring sites (hospitals/institutions with full-time faculty) and affiliate sites were recorded [9]. The list was cross-referenced with the National Resident Matching Program 2022 Program Matching Data to ensure accuracy and to exclude future residency programs [10].

Subspecialist faculty identification

Inclusion criteria for subspecialist faculty required completion of an OBGYN residency, fellowship training and/or board certification in the subspecialty, and named as faculty on program websites during the data accrual period (July 1, 2022, through August 31, 2022). A database of faculty subspecialists was created by reviewing public program websites of the sponsoring site and collecting subspecialists’ name, medical degree DO (Doctor of Osteopathic Medicine) or MD (Doctor of Medicine), residency and fellowship information. A database of additional faculty subspecialists was created by reviewing public websites of affiliate locations and recording the same information. Further fellowship training and board certification verification was done using healthgrades.com (Healthgrades Operating Company Inc., Denver, CO) [11]. Physician age was collected based on what was reported on the website [11]. Each subspecialist was hand-searched on the National Plan and Provider Enumeration System website to identify their National Provider Identifier (NPI) number to eliminate duplication in names and practice locations [12]. Physician gender was obtained from physician self-reported identification on the National Plan and Provider Enumeration System website [12]. The subspecialty faculty database was reconciled with monthly updates from the National Plan and Provider Enumeration System, resulting in 98% agreement. Subspecialties such as Addiction, Genetics, Hospital and Palliative Care Medicine, Critical Care Medicine, Menopausal and Geriatric Gynecology, and other additional training programs were excluded from the study.

Statistical analysis

The acquired data was securely stored in a password-protected database. Statistical analysis and mapping were conducted using R Statistical Software, version 4.2.2 (R Foundation for Statistical Computing, Vienna, Austria). Demographic variables were summarized using percentages or medians with interquartile ranges (IQRs) as appropriate. Continuous variables were compared using Student's t-test, while categorical and continuous data were compared using a two-way analysis of variance. Pearson's chi-square correlations were used for analyzing categorical and proportional data. A p-value of 0.05 or less was considered to be of statistical significance.

## Results

Subspecialty faculty census

**Figure 1:**
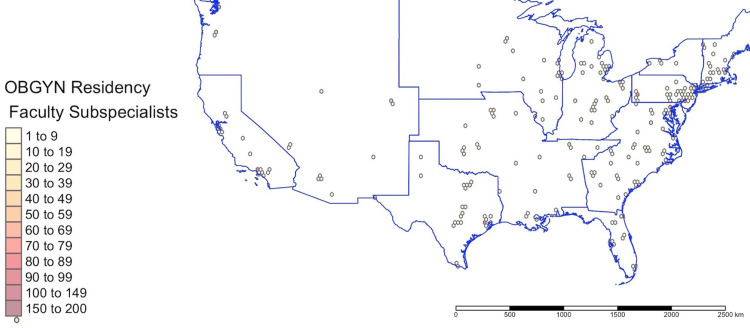
Distribution of Obstetrics and Gynecology (OBGYN) subspecialty faculty affiliated with residency programs across the continental United States Map dividing lines represent the borders of American College of Obstetricians and Gynecologists district borders.

**Figure 2:**
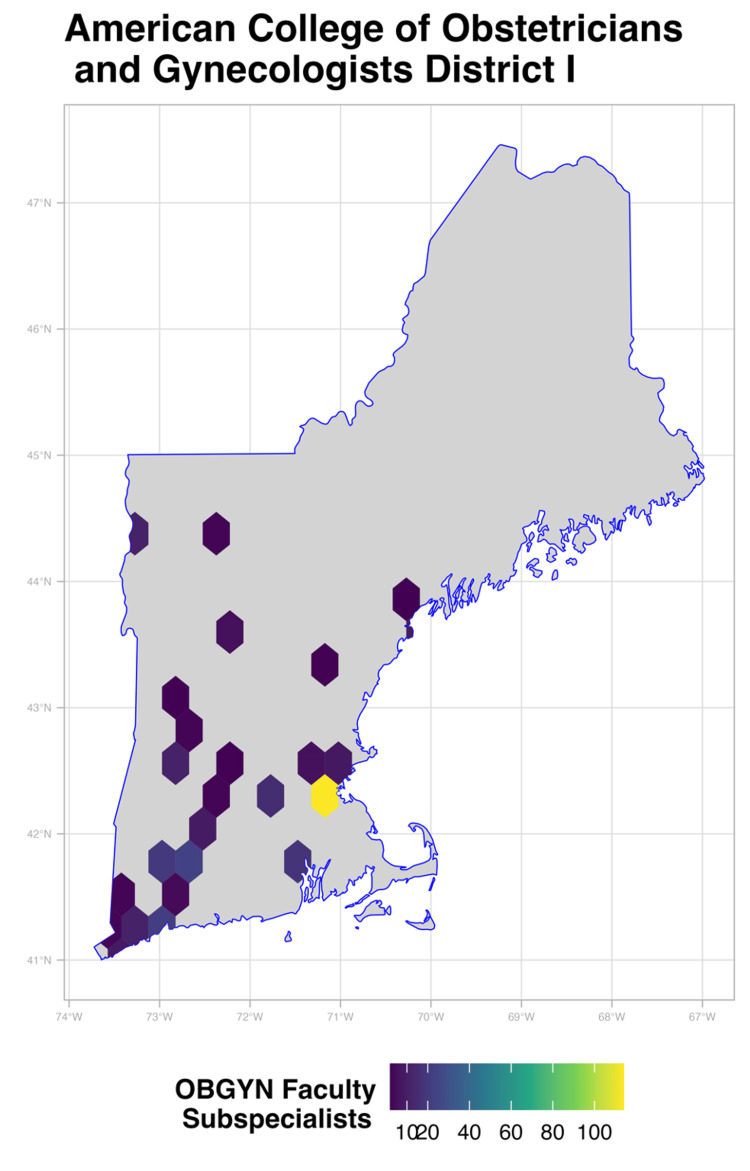
ACOG District I

**Figure 3:**
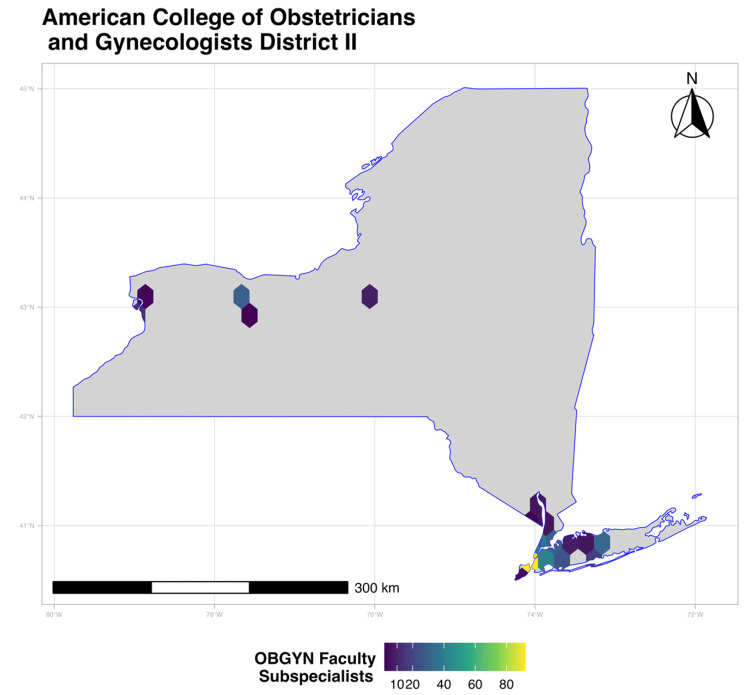
ACOG District II

**Figure 4:**
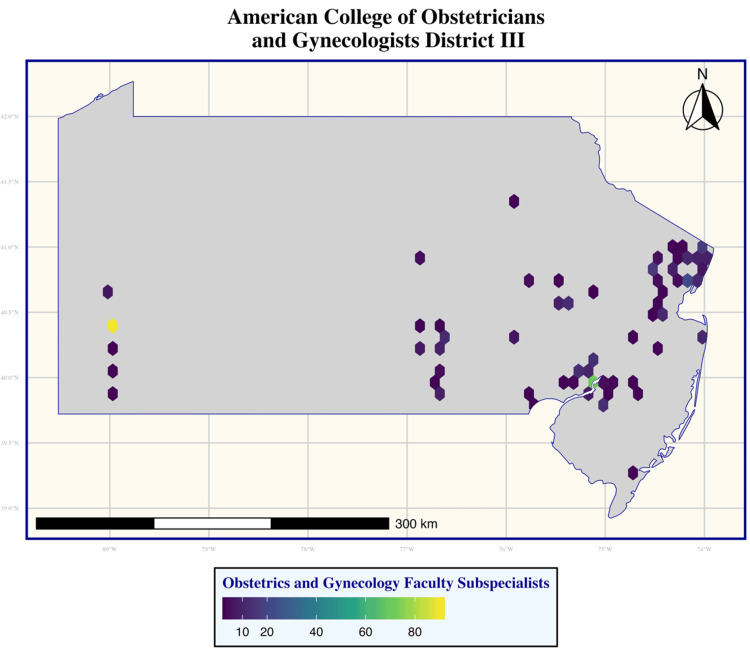
ACOG District III

**Figure 5:**
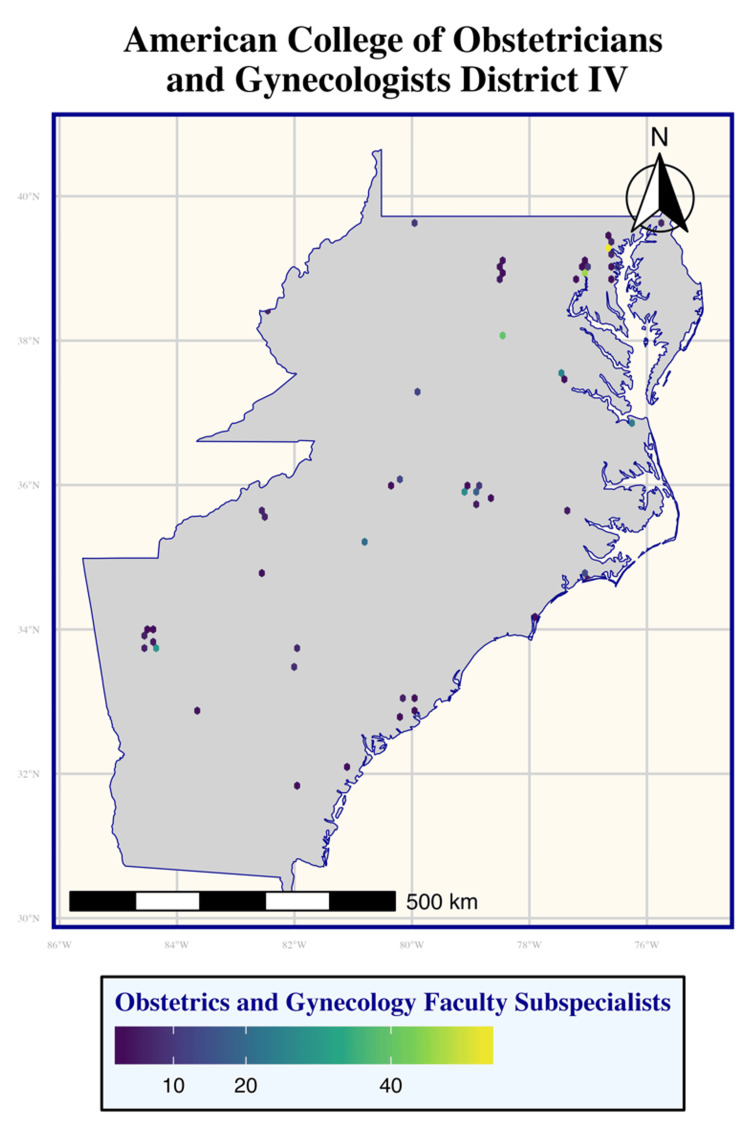
ACOG District IV

**Figure 6:**
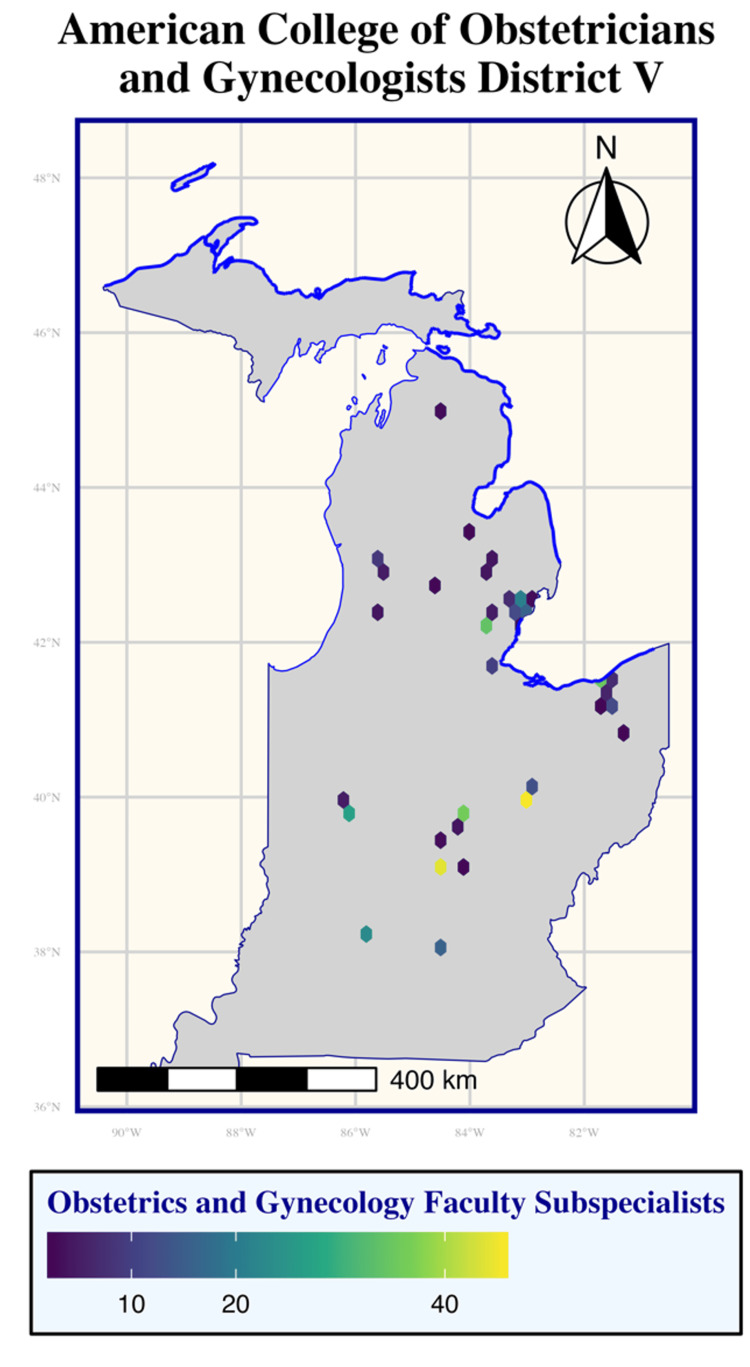
ACOG District V

**Figure 7:**
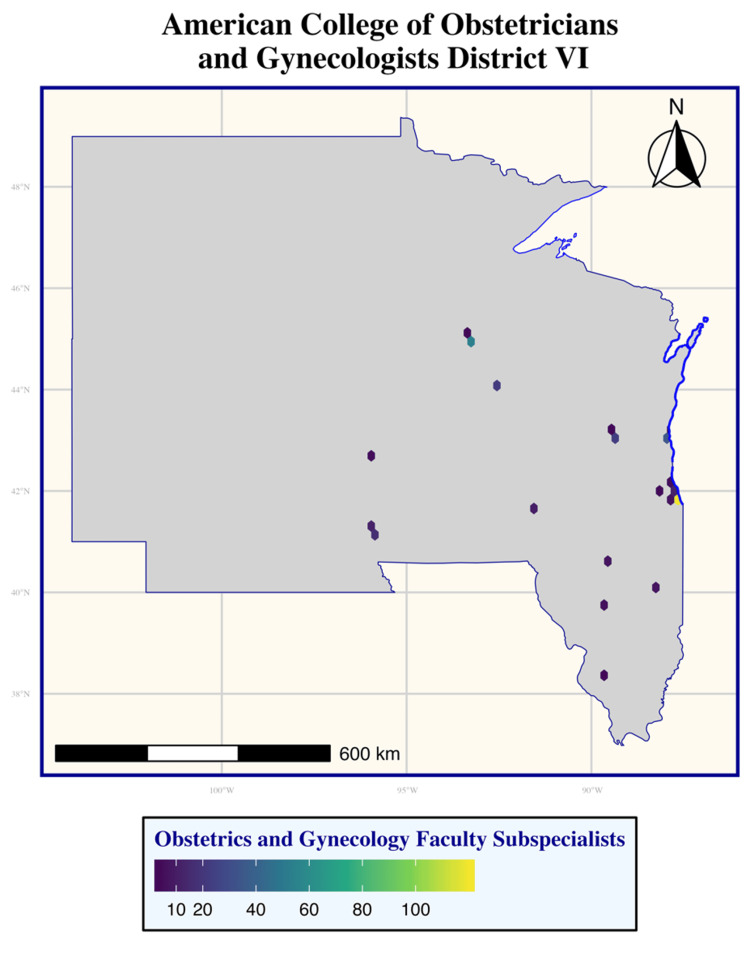
ACOG District VI

**Figure 8:**
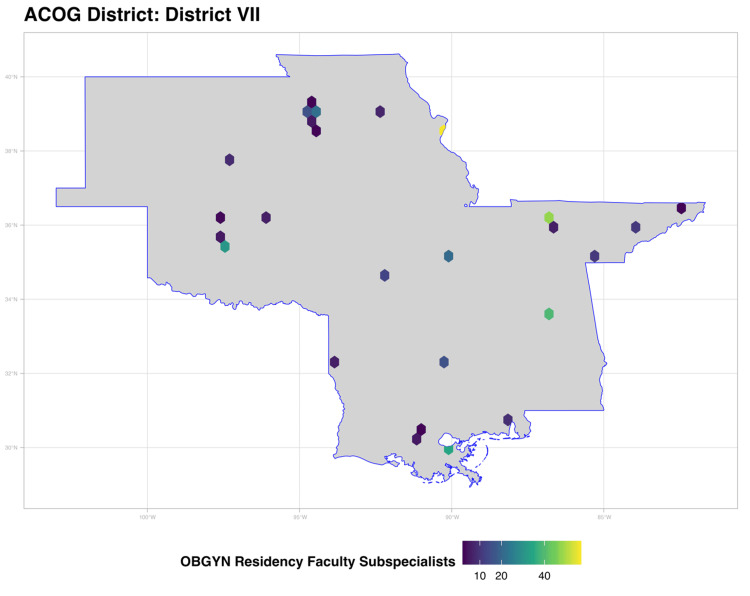
ACOG District VII

**Figure 9:**
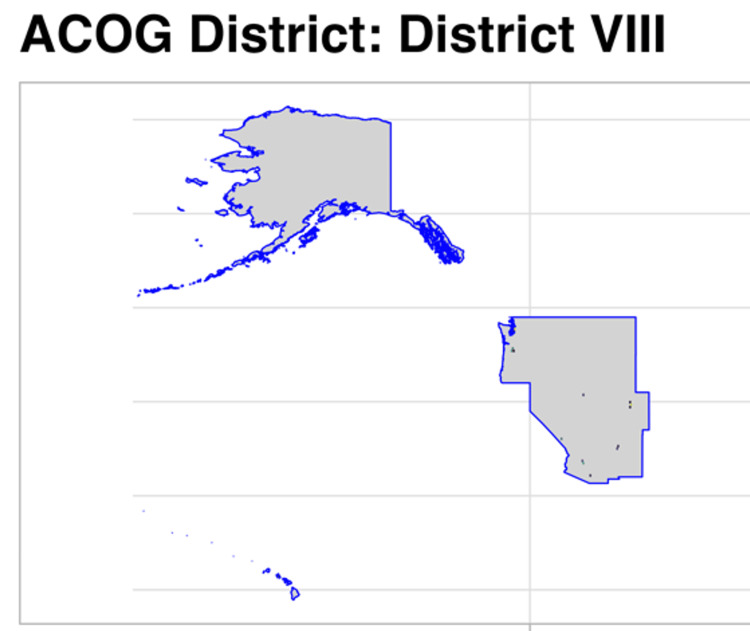
ACOG District VIII

**Figure 10:**
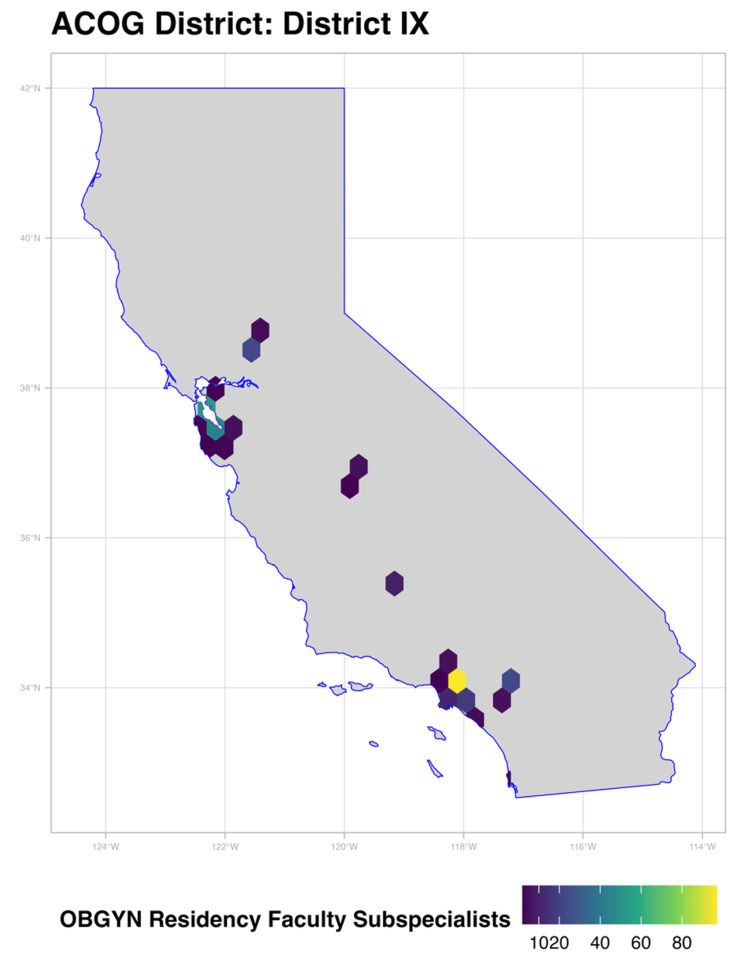
ACOG District IX

**Figure 11:**
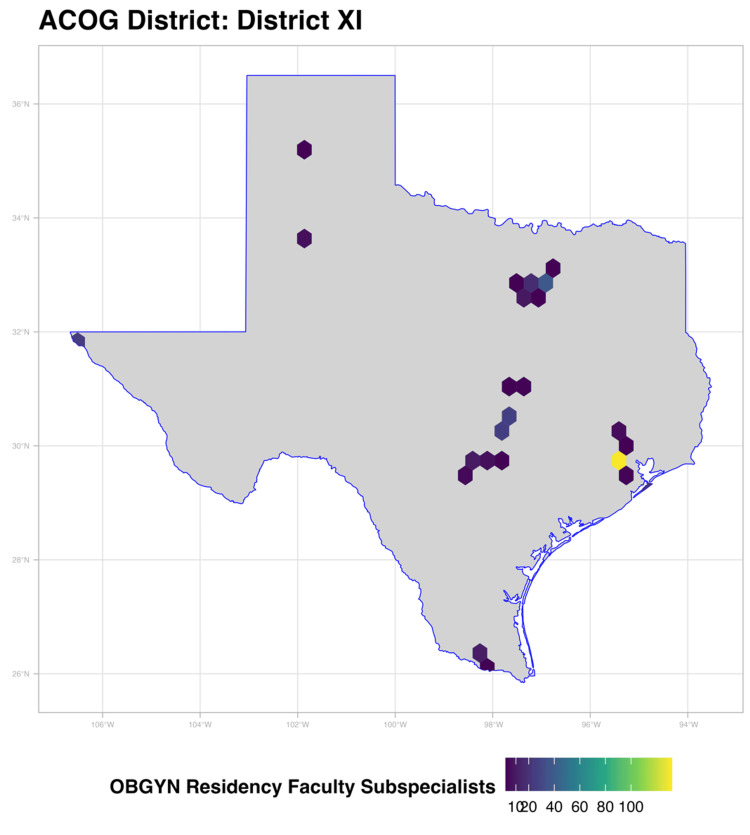
ACOG District XI

**Figure 12:**
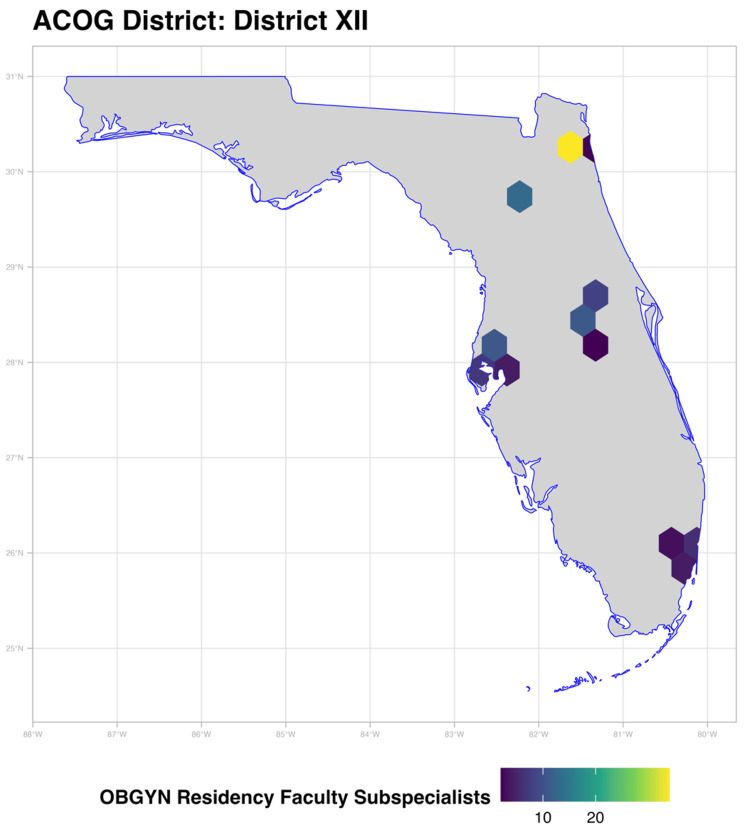
ACOG District XII

Military program (totaling seven programs) websites yielded no faculty names and were therefore excluded from the analysis. Ten civilian residency programs lacked faculty on the sponsoring site website and were excluded. Because the following states do not have a residency program, no subspecialty faculty were identified: Alaska, Idaho, Montana, North Dakota, South Dakota, and Wyoming. A total of 4,659 subspecialists were identified as faculty at 278 OBGYN residency programs. Of the identified subspecialists, 2,838 were faculty at sponsoring sites. These 2,838 and 4,659 subspecialists represented approximately 49.7% and 81.5%, respectively, of the total subspecialty workforce (n=5,716). Only 16 programs listed faculty members from all seven subspecialties on the sponsoring site website. Although the majority (59.9%) of the subspecialty workforce identified as female, male subspecialists were significantly older than female subspecialists (56 ± 10.2 versus 47 ± 9.3, p<0.01), with a greater likelihood of having greater than 10 years in practice (28 ± 9.9 versus 20 ± 9.4, p<0.01). Demographic characteristics of the 4,659 faculty subspecialists are included in Tables 1-2. Faculty subspecialists were in 45 states, the District of Columbia, and Puerto Rico. The geographic distribution is shown in Figure 1 and in the Appendices. Distribution by ACOG districts is detailed in the Appendices (Figures 2-12) [13].

**Table 1:** Characteristics of Obstetrics and Gynecology faculty subspecialists

Variable	N (%)
Age (years)	
Less than 40	712 (15.3)
40-49	1,705 (36.6)
50-59	1,238 (26.6)
60-69	833 (17.9)
70 or greater	171 (3.7)
Gender	
Female	2,793 (59.9)
Male	1866 (40.1)
Subspecialty	
Complex Family Planning	163 (3.5)
Urogynecology and Reconstructive Pelvic Surgery	682 (14.6)
Gynecologic Oncology	904 (19.4)
Maternal-Fetal Medicine	1,757 (37.7)
Minimally Invasive Gynecologic Surgery	435 (9.3)
Pediatric and Adolescent Gynecology	89 (1.9)
Reproductive Endocrinology and Infertility	629 (13.5)
Medical school degree	
Allopathic (Doctor of Medicine)	4,519 (97.0)
Osteopathic (Doctor of Osteopathic Medicine)	140 (3.0)
Medical school location	
US Senior	4,472 (96.0)
International Medical Graduate	187 (4.0)
Years in practice (from the date of medical school graduation)	
Less than 10	414 (8.9)
10-14	853 (18.3)
15 or greater	3,391 (72.8)
Residency program setting	
University-based	2,930 (62.9)
Community-based, university-affiliated	1,208 (25.9)
Community-based	521 (11.2)
American College of Obstetricians and Gynecologists (ACOG) district	
District I	308 (6.6)
District II	543 (11.7)
District III	570 (12.2)
District IV	587 (12.6)
District V	495 (10.6)
District VI	404 (8.7)
District VII	443 (9.5)
District VIII	356 (7.6)
District IX	394 (8.5)
District XI	432 (9.3)
District XII	127 (2.7)
Practice location	
Sponsor site	3,052 (65.5)
Affiliate site	1,607 (34.5)

**Table 2:** Characteristics of Obstetrics and Gynecology faculty subspecialists practicing by subspecialty CFP, Complex Family Planning; URPS, Urogynecology and Reconstructive Pelvic Surgery, GYO, Gynecologic Oncology; MFM, Maternal-Fetal Medicine; MIGS, Minimally Invasive Gynecologic Surgery; PAG, Pediatric and Adolescent Gynecology; REI, Reproductive Endocrinology and Infertility

Variable	CFP (163), N (%)	URPS (682), N (%)	GYO (904), N (%)	MFM (1,757), N (%)	MIGS (435), N (%)	PAG (89), N (%)	REI (629), N (%)	Total (4,659), N (%)	p-value
Age (years)									<0.01
Less than 40	44 (27.0)	96 (14.1)	122 (13.5)	262 (14.9)	83 (19.1)	16 (18.0)	89 (14.1)	712 (15.3)	
40-49	81 (49.7)	254 (37.2)	352 (38.9)	615 (35.0)	189 (43.4)	40 (44.9)	174 (27.7)	1,705 (36.6)	
50-59	25 (15.3)	237 (34.8)	251 (27.8)	431 (24.5)	113 (26.0)	22 (24.7)	159 (25.3)	1,238 (26.6)	
60-69	11 (6.7)	85 (12.5)	149 (16.5)	367 (20.9)	41 (9.4)	9 (10.1)	171 (27.2)	833 (17.9)	
70 or greater	2 (1.2)	10 (1.5)	30 (3.3)	82 (4.7)	9 (2.1)	2 (2.2)	36 (5.7)	171 (3.7)	
Gender									<0.01
Female	144 (88.3)	410 (60.1)	505 (55.9)	1,058 (60.2)	278 (63.9)	83 (93.3)	315 (50.1)	2,793 (59.9)	
Male	19 (11.7)	272 (39.9)	399 (44.1)	699 (39.8)	157 (36.1)	6 (6.7)	314 (49.9)	1,866 (40.1)	
Medical degree									<0.01
Doctor of Medicine	163 (100.0)	667 (97.8)	882 (97.6)	1,676 (95.4)	424 (97.5)	89 (100.0)	618 (98.3)	4,519 (97.0)	
Doctor of Osteopathic Medicine	0 (0.0)	15 (2.2)	22 (2.4)	81 (4.6)	11 (2.5)	0 (0.0)	11 (1.7)	140 (3.0)	
Medical school location									<0.01
US Senior	162 (99.4)	656 (96.2)	881 (97.5)	1,662 (94.6)	428 (98.4)	85 (95.5)	598 (95.1)	4,472 (96.0)	
International Medical Graduate	1 (0.6)	26 (3.8)	23 (2.5)	95 (5.4)	7 (1.6)	4 (4.5)	31 (4.9)	187 (4.0)	
Years in practice									<0.01
Less than 10	12 (7.4)	36 (5.3)	47 (5.2)	169 (9.6)	29 (6.7)	12 (13.5)	109 (17.3)	414 (8.9)	
10-14	37 (22.7)	136 (20.0)	169 (18.7)	318 (18.1)	95 (21.8)	16 (18.0)	82 (13.0)	853 (18.3)	
15 or greater	114 (69.9)	509 (74.7)	688 (76.1)	1,270 (72.3)	311 (71.5)	61 (68.5)	438 (69.6)	3,391 (72.8)	
Residency setting									<0.01
University-based	131 (80.4)	417 (61.1)	562 (62.2)	1,144 (65.1)	241 (55.4)	65 (73.0)	370 (58.8)	2,930 (62.9)	
Community-based, university-affiliated	24 (14.7)	187 (27.4)	228 (25.2)	438 (24.9)	147 (33.8)	19 (21.3)	165 (26.2)	1,208 (25.9)	
Community-based	8 (4.9)	78 (11.4)	114 (12.6)	175 (10.0)	47 (10.8)	5 (5.6)	94 (14.9)	521 (11.2)	
American College of Obstetricians and Gynecologists district									<0.01
District I	12 (7.4)	55 (8.1)	50 (5.5)	116 (6.6)	28 (6.4)	8 (9.0)	39 (6.2)	308 (6.6)	
District II	35 (21.5)	67 (9.8)	92 (10.2)	187 (10.6)	75 (17.2)	3 (3.4)	84 (13.4)	543 (11.7)	
District III	12 (7.4)	84 (12.3)	91 (10.1)	206 (11.7)	55 (12.6)	5 (5.6)	117 (18.6)	570 (12.2)	
District IV	21 (12.9)	82 (12.0)	120 (13.3)	223 (12.7)	36 (8.3)	21 (23.6)	84 (13.4)	587 (12.6)	
District V	0 (0.0)	76 (11.1)	103 (11.4)	204 (11.6)	47 (10.8)	7 (7.9)	58 (9.2)	495 (10.6)	
District VI	12 (7.4)	79 (11.6)	88 (9.7)	149 (8.5)	33 (7.6)	4 (4.5)	39 (6.2)	404 (8.7)	
District VII	4 (2.5)	74 (10.9)	83 (9.2)	166 (9.4)	43 (9.9)	13 (14.6)	60 (9.5)	443 (9.5)	
District VIII	36 (22.1)	37 (5.4)	67 (7.4)	134 (7.6)	38 (8.7)	10 (11.2)	34 (5.4)	356 (7.6)	
District IX	29 (17.8)	57 (8.4)	94 (10.4)	146 (8.3)	29 (6.7)	6 (6.7)	33 (5.2)	394 (8.5)	
District XI	0 (0.0)	50 (7.3)	84 (9.3)	177 (10.1)	40 (9.2)	12 (13.5)	69 (11.0)	432 (9.3)	
District XII	2 (1.2)	21 (3.1)	32 (3.5)	49 (2.8)	11 (2.5)	0 (0.0)	12 (1.9)	127 (2.7)	
Practice location									<0.01
Sponsor site	143 (87.7)	472 (69.2)	610 (67.5)	1226 (69.8)	333 (76.6)	73 (82.0)	195 (31.0)	3052 (65.5)	
Affiliate site	20 (12.3)	210 (30.8)	294 (32.5)	531 (30.2)	102 (23.4)	16 (18.0)	434 (69.0)	1607 (34.5)	

Complex Family Planning subspecialists

A total of 163 CFP subspecialists were identified in 19.0% of residency programs (n=59). All (100.0%) CFP faculty subspecialists had allopathic medical degrees. CFP subspecialists were the youngest of all subspecialty faculty (27.0% less than 40 years old), but had a similar number of years of experience to other subspecialists (69.9% had a practice experience of 15 years or more after their medical school graduation date). They were most likely of any faculty subspecialist to be employed at a university-based residency program (80.4%) and were most likely to practice at a sponsoring site (87.7%). CFP faculty were significantly more common in university-based residencies compared to community-based or community-based university-affiliated residencies (p=0.003). ACOG District VIII (Alaska, Arizona, Colorado, Hawaii, Idaho, Montana, Nevada, New Mexico, Oregon, Utah, Washington, and Wyoming) contained the highest number of CFP faculty (n=36). CFP faculty were the most geographically skewed subspecialists across states. There were 20 states without CFP faculty, which was the least coverage compared to all other subspecialty faculty.

Gynecologic Oncology subspecialists

GYO faculty subspecialists were the most experienced faculty members, with 76.1% having a practice experience of 15 years or more after their medical school graduation date. Results revealed a notable correlation between MFM and GYO, with a correlation coefficient of 0.44, suggesting that in programs where MFM is present, there is a tendency for GYO to be present, and vice versa. GYO faculty were the most geographically widespread subspecialists as they were present in 45 states.

Maternal-Fetal Medicine subspecialists

MFM faculty subspecialists were the most plentiful (n=1,757), with 90.3% of residency programs listing at least one MFM faculty member. Among programs with at least one subspecialist, MFM subspecialists were the most common, with a median of 5 (IQR 1-33) subspecialists per program. Of the subspecialties, they were the least allopathic-dominated subspecialty (4.6% of MFM faculty have a Doctor of Osteopathic Medicine; p<0.001) and had the most International Medical Graduates (5.4%).

Minimally Invasive Gynecologic Surgery subspecialists

There was a correlation between MIGS and REI subspecialists with a correlation coefficient of 0.21. The geographic distribution of MIGS subspecialists showed a high concentration in District II (New York) and a low concentration in District XII (Florida). MIGS subspecialists were the most common faculty in university-based settings compared to all other subspecialties (p<0.001).

Pediatric and Adolescent Gynecology subspecialists

A PAG subspecialist was listed by the fewest number of residency programs (n=53, 17.7%). The PAG faculty subspecialty was the most female-dominated subspecialty (only 6.7% males) and was the least geographically diverse of all faculty subspecialists. Our search found no PAG faculty subspecialist present in District XII (Florida).

Reproductive Endocrinology and Infertility subspecialists

The REI faculty subspecialty was the most male-dominated (49.9%). REI subspecialists were the oldest, with 32.9% practicing beyond the median physician retirement age of 64 years (IQR 59-69).13 The median age of REIs was 53 years (IQR 43-62), compared to 48.5 years for all other subspecialties (IQR 42-57, p<0.001). They were the only subspecialty where most faculty did not practice at the sponsoring site (69.0% practiced at an affiliate site).

Urogynecology and Reconstructive Pelvic Surgery subspecialists

URPS faculty subspecialists had the lowest proportion of faculty aged 70 years or older (p<0.001). ACOG District VI (Illinois, Iowa, Minnesota, Nebraska, North Dakota, South Dakota, Wisconsin) had more urogynecologists represented than any other subspecialty. There were no differences in age, gender, or geographic distribution between urogynecology faculty and other faculty subspecialists (p>0.05).

## Discussion

Our study identified that 81.5% of the total OBGYN subspecialty workforce was involved in the training of residents, with 49.7% of the faculty workforce at the residency program sponsoring site. The subspecialty faculty workforce was primarily female, mid-career, and had an allopathic degree. Male subspecialists were more senior than their female counterparts. University-based programs had a higher concentration of subspecialty faculty than community-based programs, and the distribution of faculty across ACOG districts was relatively even.

The OBGYN subspecialty workforce analysis in 2017 demonstrated an increasing trend in subspecialization, with one out of four residents pursuing subspecialty training and one in three expected to pursue subspecialty training by 2020 [8]. Among residents, a subspecialist mentor is an essential factor in deciding to pursue fellowship [7]. Additionally, the ACGME requires a designated faculty member for each subspecialty [6]. Yet, only 16 residency programs listed board-certified and/or fellowship-trained subspecialists in all seven subspecialty faculty positions on their sponsor site website. This is a surprising finding and implies that much of the training residents receive is by a designated generalist that is not subspecialty trained. This is especially the case for subspecialties that have less subspecialists per residents: Complex Family Planning, Minimally Invasive Gynecologic Surgery, and Pediatric and Adolescent Gynecology. Even though the majority of subspecialists are involved in resident training and education, it appears the majority of residencies lack exposure to subspecialty-trained practitioners in all subspecialties. How residents train, and with whom, affects resident career planning and patient care.

Subspecialists are vital in expert resident education and complex patient care, and their availability to trainees and patients is paramount. There are currently many ongoing efforts to bridge the gap between subspecialty training and trainees. The ACGME requirement of a qualified professional dedicated to resident education in each subspecialty is indeed a start. Also, the number of residents pursuing fellowship and the number of fellowship spots are increasing, which will likely increase the academic subspecialty workforce. Although the majority of subspecialists are practicing at sponsor sites, affiliate sites are an important way to expose residents to subspecialty training. Additionally, there are opportunities for residents to rotate at other residency programs to receive subspecialty training opportunities that their home programs lack.

Despite the geographic diversity of subspecialists identified in our study, increasing geographic disparities are still present. Most subspecialists are faculty and geographically affiliated with a residency training program, and residency training programs tend to be concentrated in metropolitan areas, partially explaining the shortage of rural subspecialty access [8]. While increasing rates in subspecialization contribute to a more robust subspecialist workforce, this trend inherently decreases the ratio of generalists entering the field of OBGYN [8]. Our study noted REI subspecialists are more senior than other faculty subspecialists and are nearing retirement age [14]. This workforce gap would further exacerbate the availability of subspecialist mentorship in residencies and underserved patient populations, not to mention put stress on the remaining workforce.

While prior workforce analyses have quantified subspecialists and described demographics, this is the most recent study to quantify the faculty workforce. This study is limited by publicly available data and relies on the accuracy of residency program websites. We attempted to maximize website information accuracy by cross-referencing board certification with NPI numbers and physician age with the Healthgrades site [11-12]. The generalizability of the study is limited by the exclusion of military residency programs and subspecialty faculty and also by the exclusion of subspecialists who did not complete an OBGYN residency (namely, urologists working on faculty as urogynecologists) and/or fellowship training in a subspecialty other than CFP, GYO, MFM, MIGS, PAG, REI or URPS. Although we attempted to provide a comprehensive census of the subspecialty faculty workforce by including subspecialists listed on affiliate site websites, it is quite possible that residents work with private practice physicians that are not accounted for on either the primary site website or the affiliate site websites.

## Conclusions

This study quantified the Obstetrics and Gynecology subspecialty workforce involved in the clinical education of OBGYN residents. The subspecialties involved in the analysis included Complex Family Planning, Gynecologic Oncology, Maternal-Fetal Medicine, Minimally Invasive Gynecology, Pediatric and Adolescent Gynecology, Reproductive Endocrinology and Infertility and Urogynecology. Most of the OBGYN subspecialty workforce is involved in the clinical education of Obstetrics and Gynecology residents. Half of the total subspecialty workforce is on faculty at a residency program sponsor site. This study also described the demographics of the OBGYN subspecialists working with residents. The subspecialty faculty workforce is primarily female, has an allopathic degree, is mid-career, and is geographically diverse. The REI faculty workforce is closer to retirement age than other subspecialty faculty and there is a paucity of subspecialty faculty in ACOG District XII (Florida). This is the most recent OBGYN subspecialist census and the only workforce analysis to focus on subspecialist faculty. The results impact residency programs as they work to meet ACGME requirements and recruit residents, residents as they train and plan their career and patients as they access subspecialty care.

## Appendices

ACOG districts and sections [14]

District I: Atlantic Provinces, Chile, Connecticut, Maine, Massachusetts, Quebec, Rhode Island, and Vermont

District II: New York and Bermuda

District III: Delaware, Dominican Republic, New Jersey, and Pennsylvania

District IV: District of Columbia, Georgia, Maryland, North Carolina, South Carolina, Virginia, West Virginia, Argentina, Puerto Rico and the West Indies

District V: Indiana, Kentucky, Ohio, Michigan, and Ontario

District VI: Illinois, Iowa, Minnesota, Nebraska, North Dakota, South Dakota, Wisconsin, Manitoba, Saskatchewan, and Peru

District VII: Alabama, Arkansas, Kansas, Louisiana, Mexico, Mississippi, Missouri, Oklahoma, and Tennessee

District VIII: Alaska, Alberta, Arizona, British Columbia, Central America, Colorado, Hawaii, Idaho, Montana, Nevada, New Mexico, Oregon, Utah, Washington, Wyoming, American Samoa, Guam, Northwest Territory, and Yukon Territory

District IX: California and Ecuador

District X: Armed Forces

District XI: Texas

District XII: Florida and Columbia

ACOG district maps
